# Ultrasonic Neuromodulation and Sonogenetics: A New Era for Neural Modulation

**DOI:** 10.3389/fphys.2020.00787

**Published:** 2020-07-16

**Authors:** Songyun Wang, Weilun Meng, Zhongyuan Ren, Binxun Li, Tongjian Zhu, Hui Chen, Zhen Wang, Bo He, Dongdong Zhao, Hong Jiang

**Affiliations:** ^1^Department of Cardiology, Renmin Hospital of Wuhan University, Wuhan, China; ^2^Department of Cardiology, Shanghai Tenth People’s Hospital, Tongji University School of Medicine, Shanghai, China; ^3^Medical Department, Nanjing Medical University, Nanjing, China; ^4^Medical Department, Soochow University Medical College, Suzhou, China

**Keywords:** ultrasound, neuromodulation, sonogenetics, molecular biology, mechanism

## Abstract

Non-invasive ultrasonic neural modulation (UNM), a non-invasive technique with enhanced spatial focus compared to conventional electrical neural modulation, has attracted much attention in recent decades and might become the mainstream regimen for neurological disorders. However, as ultrasonic bioeffects and its adjustments are still unclear, it remains difficult to be extensively applied for therapeutic purpose, much less in the setting of human skull. Hence to comprehensively understand the way ultrasound exerts bioeffects, we explored UNM from a basic perspective by illustrating the parameter settings and the underlying mechanisms. In addition, although the spatial resolution and precision of UNM are considerable, UNM is relatively non-specific to tissue or cell type and shows very low specificity at the molecular level. Surprisingly, Ibsen et al. (2015) first proposed the concept of sonogenetics, which combined UNM and mechanosensitive (MS) channel protein. This emerging approach is a valuable improvement, as it may markedly increase the precision and spatial resolution of UNM. It seemed to be an inspiring tool with high accuracy and specificity, however, little information about sonogenetics is currently available. Thus, in order to provide an overview of sonogenetics and prompt the researches on UNM, we summarized the potential mechanisms from a molecular level.

## Introduction

Harvey (1929) fortuitously observed the excitation of peripheral nerves by ultrasound (US), which is the first time UNM was reported. Several decades later, studies showed that FUS could propagate across the skull and efficiently modulate the activity of mammalian brain tissue *in vitro* and *in vivo* (Mueller et al., 2014; Tyler et al., 2018). UNM has been utilized in a variety of clinical situations, such as an analgesic strategy for cancer-related and neuropathic pain, neurosurgery for Parkinson’s disease or essential tremor, urological surgery to ablate renal cell carcinoma or prostate cancer, and thrombolysis within cerebral vessels (Dobrakowski et al., 2014; Klatte et al., 2014; Strauss et al., 2014; Ahmed et al., 2015). Owning to its non-invasive property and enhanced spatial focus, UNM has emerged as a promising non-invasive UNM approach and has the potential to treat neurological disorders. However, neither the parameter settings nor the bioeffects of UNM has been well-understood, which impeded the application of UNM therapies.

More importantly, although the spatial resolution and precision of UNM are considerable, US is relatively non-specific to tissue or cell type and shows very low specificity at the molecular level. Recently, optogenetics and magnetogenetics, which combine optics or magnetics with genetics, were proposed and proven to be novel approaches for cellular specific neuromodulation (Aston-Jones and Deisseroth, 2013; Monzel et al., 2017). However, both methods have difficulty in delivering stimuli to targeted regions of neurons located in the deeper brain (Fregni and Pascual-Leone, 2007). Surprisingly, Ibsen et al. (2015) firstly combined ultrasonic with genetics in controlling the behavior of *C. elegans* in 2015. The study showed that US could elicit cell-specific excitation in ultrasonically sensitized objects. Afterward, the concept of ‘sonogenetics’ was proposed. Sonogenetics, which focuses on the genetic modulation of US-sensitive neurons and their specific responses to US via the expression of MS receptors, has non-invasive property and enhanced spatial focus, which might be a better choice for specific neural modulation than optogenetics and magnetogenetics. However, as a novel concept, little information was known about sonogenetics. A comprehensive review of sonogenetics studies is warranted to provide a clue for further researches.

## Ultrasound Parameters

**FIGURE 1:**
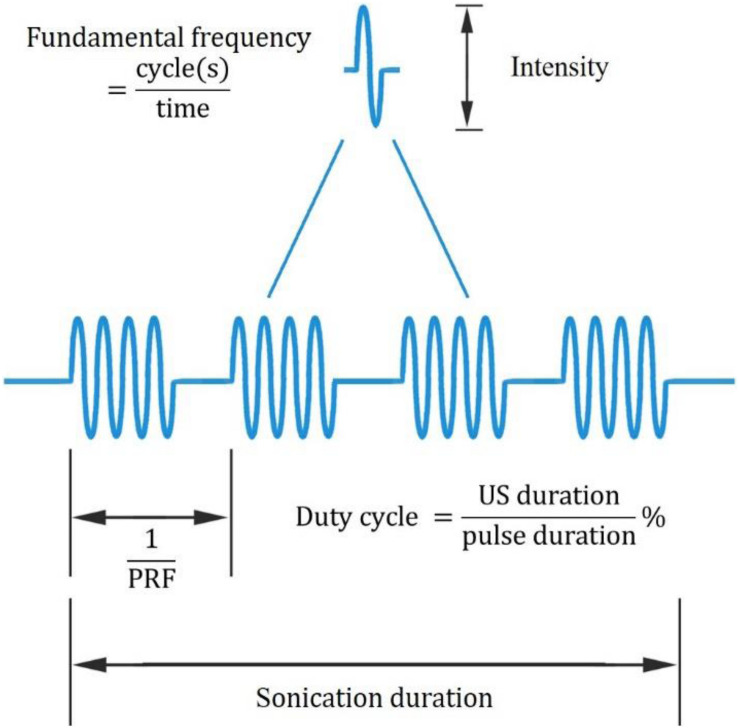
Ultrasonic parameters. PRF denotes pulse repetition frequency, the duration of 1 complete pulse.

In recent years, UNM, as a non-invasive neural modulation approach with enhanced spatial accuracy, has attracted more and more attentions and lots of studies have been done to optimize the method. From basic aspect, US is acoustic wave with frequency over 20 thousand Hertz, and propagates primarily in longitudinal and transverse waveforms. As other sound waves, US is interactive that can be either superimposed or counteracted. Through phase array modification of multiple US transducers, US energy can be delivered and concentrated at a certain spatial focus, which is recognized as FUS. Current studies show that both unfocused and focused US can modulate neural activities, however, the delivery pattern and modulative effect differ from each other. On one hand, unfocused US requires higher frequency and longer duration to achieve therapeutic effect (Hameroff et al., 2013; Gibson et al., 2018), while FUS delivers energy with more efficiency (Lee et al., 2015). On the other hand, the impact of unfocused US on targeted site differs from that of FUS. Studies show that when US delivered at primary sensory cortex, unfocused US increases neural activity (Gibson et al., 2018) while FUS is inhibitory (Legon et al., 2018b). Among various factors modulating neural modulative effect, adjusting US parameters plays an important role. Thus, a comprehensive understanding of US parameters is needed. Ultrasonic parameters primarily include intensity (I), FF, duration, DC and PRF (Tables 1, 2), as illustrated in Figure 1.

**TABLE 1:** Ultrasonic parameter settings in human studies.

	Study	Target	Ultrasonic parameters				
			
			Intensity (W/cm^2^)	Frequency (MHz)	Duration	Duty cycle (%)	PRF (kHz)
**Excitatory**	Legon et al. (2014)	Primary somatosensory cortex	ISPPA 5.9	0.5	500 ms	36	1
	Lee et al. (2015)	Primary somatosensory cortex	ISPPA 3 ISPTA 0.7	0.25	300 ms	50	0.5
	Lee et al. (2016a)	Primary and secondary somatosensory cortex	ISPTA 3.5–4.4 ISPPA 7–8.8	0.21	500 ms	50	0.5
	Lee et al. (2016b)	Visual cortex	ISPPA 0.7–6.6	0.27	300 ms	50	0.5
	Ai and Xiong (2016)	Primary sensorimotor cortex; caudate area	ISPPA 6	0.5, 0.86	500 ms	36,50	1,0.5
	Monti et al. (2016)	Thalamus	ISPTA 0.72	0.65	3 s * 10 times	5	0.1
	Ai et al. (2018)	Primary motor cortex	ISPPA 16.95	0.5	500 ms	36	1
	Gibson et al. (2018)	Primary motor cortex	ISPPA 24.96 ISPTA 132.85	2.32	2 min	<1	/
**Inhibitory**	Hameroff et al. (2013)	Inferolateral frontal lobe	ISPTA 0.152	8	15 s	100	/
	Legon et al. (2018a)	Primary motor cortex	ISPTA 6.16	0.5	500 ms	36	1
	Legon et al. (2018b)	Thalamus	ISPPA 7.02	0.5	300 ms every 4 s	36	1

**TABLE 2:** Ultrasonic parameter settings in animal studies.

	Study	Animal	Target	Ultrasonic parameters				
				
				Intensity (W/cm^2^)	Frequency (MHz)	Duration	Duty cycle (%)	PRF (kHz)
**Excitatory**	Tufail et al. (2010)	Mice	Motor cortex	0.075–0.229 ISPPA	0.25–0.5	26–333 ms	19–86	1.2–3
	Li et al. (2019)		Primary somatosensory cortex	46 ISPPA 0.7 ISPTA	2	300 ms	30	1
	Xie et al. (2018)		Primary motor cortex	1.10 AI	0.5	ND	ND	1
	Sato et al. (2018)		Primary somatosensory cortex, primary auditory cortex and visual cortex;	0.034–4.2 ISPTA	0.5	80 ms	ND	1.5
	Kim et al. (2015)	Rats	Visual area	5 ISPPA	0.35	150 s	8.3	0.1
	Yu et al. (2016)		Multiple sites	0.01 ISPTA	0.5	5 or 200 ms	ND	2
	Sharabi et al. (2019)		Medulla oblongata region	27.2 ISPPA	0.23	100 ms	100	1
	Yoo et al. (2011b)		Thalamus	3.3 or 6 ISPPA	0.65	ND	5	0.1
	Kim et al. (2014b)		Motor cortex	4.9–5.6 ISPPA	0.35	300 ms	50	0.06–2.8
	Zhang et al. (2019)		Prefrontal cortex	7.59 ISPPA	0.5	400 ms for 15 min per day for 2 weeks	60	1.5
	Yoo et al. (2011a)	Rabbits	Motor cortex	12.6 ISPPA	0.69	500, 1,000, 1,500, and 2,000 ms	50	0.01
	Lee et al. (2015)	Ovine	Sensorimotor cortex	6.9 ISPPA	0.25	300 ms	50	0.5
	Yoon et al. (2019)		Left M1 anterior to central sulcus/left thalamus	15.8 18.2 ISPPA 4.7–12.7/15.8–18.2 ISPTA	0.25	60–200 ms	30–100	100–1000
	Yang et al. (2018)	Macaque monkey	Primary somatosensory cortex	29.5 ISPPA	0.25	300 ms * 10	50	2
	Wattiez et al. (2017)		Frontal eye field	1.9–5.6 ISPPA	0.32	100 ms	100	/
	Guo et al. (2018)	Guinea pigs	Primary somatosensory cortex, primary auditory cortex and visual cortex	0.02 ISPPA	0.22	500 ms	ND	1
**Inhibitory**	Kim et al. (2015)	Rats	Visual area	3 ISPPA	0.35	150 s	5	0.1
	Chu et al. (2015)		Left primary somatosensory cortex	0.55, 0.8 MI	0.4	120 s	1	0.01
	Kim et al. (2014a)		Motor cortex	3 ISPTA	0.35	300 ms	50	1
	Sharabi et al. (2019)		Medulla oblongata region	27.2 ISPPA	0.23	100 ms	100	1
	Daniels et al. (2018)		Inferior colliculus	2.3, 4.6 ISPPA	0.23	100 ms	3	1
	Yoo et al. (2011a)	Rabbits	Visual area	3.3 ISPPA	0.69	>7,000 to 8,000 ms	5	0.1
	Yoon et al. (2019)	Ovine	Left S1 posterior to central sulcus/left thalamus	5.4 11.6 ISPPA 0.16–0.58 ISPTA	0.25	/	3–5	0.05–0.1
	Dallapiazza et al. (2018)	Yorkshire swine	Sensory thalamus; ventroposterolateral thalamic nucleus	25–30 ISPPA	1.14	500 ms	43.7	0.01
	Daniels et al. (2018)	Pig	Auditory cortex region	2.3, 4.6 ISPPA	0.23	100 ms	3	1

### Intensity (I)

Intensity is the acoustic energy generated by US, usually described as spatial-peak pulse-average intensity (I_*SPPA*_) or spatial-peak temporal-average intensity (I_*SPTA*_) for application. Intensity is a major determinant of US bioeffects. Based on intensity levels, FUS can be categorized as HIFU or LIFU. HIFU is defined as US with typical intensity levels ranging from approximately 100 W/cm^2^ to 10000 W/cm^2^ (Quadri et al., 2018). HIFU was used in early neuromodulation research, while later on, it is more commonly used for ablative surgery to minimize surgical trauma (Klatte et al., 2014). Under high intensity, US exerts bioeffects through thermal elevation and coagulative necrosis, which could lead to irreversible tissue damage. These biological effects were proven to be therapeutic in the surgical treatment of Parkinson’s disease (Strauss et al., 2014) and essential tremor (Dobrakowski et al., 2014; Ahmed et al., 2015). LIFU, with an intensity less than 3 W/m^2^, could reversibly modulate local tissue under controlled temperatures (Rezayat and Toostani, 2016). This modulatory effect of LIFU is highly intensity dependent. Velling and Shklyaruk (1988) found no detectable effect of LIFU on the brain at low (less than 0.1 mW/cm^2^) intensities in cats and rabbits. As the intensity increased to 1 to 100 mW/cm^2^, electrical activity was recorded by US stimulation. However, US with higher intensity from 1 to 100 W/cm^2^ suppressed brain activity. Therefore, the neural modulatory effect of LIFU is bidirectional, and intensity plays a crucial role.

### Fundamental Frequency (FF)

The FF is the oscillation cycles per unit time. The application of US depends on the FF to a great extent. High frequency (1–20 MHz) US is adopted for diagnostic intentions, medium frequencies (0.7–3 MHz) are used for therapeutic use, and low frequencies (20–200 kHz) are utilized in industry (Ahmadi et al., 2012). FF determines the penetration property, which accounts for the spatial resolution of US. Theoretically, as FF is inversely proportional to wavelength, US with higher FF enables a tighter and deeper focus, resulting in higher space resolution. However, as FF increases, energy simultaneously attenuates and is converted to a large amount of heat and dispersed to surrounding tissues. This attenuation and energy conversion render high FF US less efficient, and the thermal effect could destroy local tissue (Legon et al., 2018a). Meanwhile, the efficiency of US can be optimized by adjusting the FF within the appropriate range. A study proved that from 250 kHz to 600 kHz, stimulation efficiency increases with lower FF (King et al., 2013). Another study showed that from 0.3 to 2.9 MHz, the higher the frequency that was adopted, the higher the spatial peak intensity required to maintain equal efficiency (Foley et al., 2007). Therefore, a relatively lower FF is suggested for optimizing the neural modulatory effect.

### Duration

The duration of US application is defined as the total time from the beginning of the first pulse to the end of the last pulse. Evidence from preclinical studies suggests that longer durations of LIFU application favor the inhibition of cortical neurons, whereas short durations produce excitation (Kim et al., 2014a; Plaksin et al., 2016). Additionally, a study proved that a longer term (>10 s) application of LIFU (10–100 mW/cm^2^) can induce the relatively longer-term alteration of neural activity (Velling and Shklyaruk, 1988). Hence, application duration affects the duration of US effect on neural activity, either excitatory or inhibitory.

### Duty Cycle (DC)

The DC dictates the proportion of US cycles in each pulse. Based on DC, US can be delivered in a continuous manner without interruption (DC = 100%) or in a pulsing manner where intervals exist (DC < 100%). Although continuous application has been proven to elicit neural activation (Kim et al., 2014a), pulsing application delivery induced safer and more efficient neural activation in most studies. Moreover, the optimal DC could minimize the neural activation threshold, and DC is considered to be an important variable in US neural modulation (Plaksin et al., 2016).

### Pulse Repetition Frequency (PRF)

Pulse repetition frequency (PRF) describes the number of pulses delivered per unit time at FF. Based on experimental results, the modulation of neurons correlates with PRF. Necessarily, the inhibitory effect of US can only be achieved when PRF is above 100 Hz. As PRF increases beyond 500 Hz, US stimulates neuron activity with evoked EEG (Kim et al., 2014a).

## Bioeffect of US

### Thermal Effect (Figure 2A)

As US propagates in an attenuating pattern due to absorption and scattering, the lost energy subsequently converts to heat. Once the amount of heat generated exceeds that the amount of heat dissipated, the temperature of local tissue increases, a property described as the thermal effect of US. The thermal effect is well understood, as illustrated by computer modeling (Pinton et al., 2010; Rossmanna and Haemmerich, 2014), and it is known to alter neural function by means of reversibly decreasing synaptic transmission or irreversibly protein denaturation (Rossmanna and Haemmerich, 2014). This neural modulation by heat generation primarily depends on the frequency, intensity, duration, sound speed, and density of tissue, as described by a formula by Nyborg in 1981 (O’Brien, 2007). The thermal effect of US can be either deleterious or therapeutic. On the one hand, through the non-invasive rapid delivery of thermal energy, HIFU could ablate diseased tissue with fewer side effects. Guided by MRI, focal thermal ablation of deep-brain circuits was proven to be beneficial for movement disorders or psychiatric disease (Dougherty et al., 2015). In addition, even though the temperature elevation is less prominent in LIFU, subtle temperature changes in the range of physiological conditions can modulate neural activity. Temperature influences the bioactivity of organelles and chemical transmission at synapse junctions (Fry, 1954). Through adjusting US parameters, heat generation by LIFU elicits cellular activation under acute exposure, while the prolonged exposure induces inhibitory cellular effects (Hakimova et al., 2015). On the other hand, when used to modulate physiological function, such as applying LIFU for neural modulation, the overheating caused by US can result in irreversible damage to local tissue and is considered a major side effect. For safety concerns, the American Institute of Ultrasound in Medicine (AIUM) stipulated the upper limits of US parameters for *in vivo* mammalian experiments to avoid overheating as follows: intensity < 100 mW/cm^2^ and exposure time < 500 s for unfocused US and intensity < 1 W/cm^2^ and exposure time < 50 s for FUS (O’Brien, 2007). As it has dual effects, the thermal effect should be thoroughly evaluated with intensive monitoring during UNM.

**FIGURE 2:**
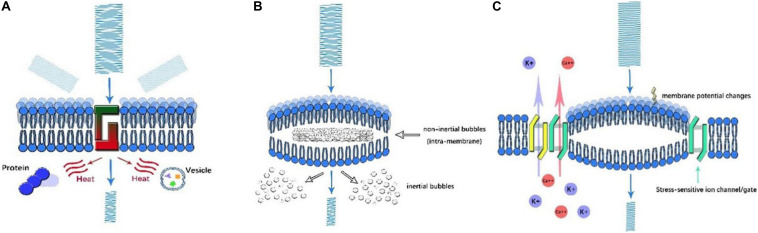
Illustration of ultrasonic mechanisms of neural modulation. **(A)** Thermal effect. As US propagates through the cell membrane, the amplitude of US decreases as its energy is converted into heat. By scattering and absorption, the denaturation of intracellular proteins and a decrease in synaptic transmission occur, which affect neural activity. **(B)** Cavitation. Once high-frequency US is applied, microbubbles are generated in the presence of gas. Based on the motion of microbubbles, inertial and non-inertial microbubbles can be identified. Intramembrane inertial microbubbles could alter membrane potential, while the rupture of non-inertial microbubbles leads to local organelle damage. **(C)** Acoustic radiation forces. As US encounters membranes, ultrasonic energy is transformed into mechanical forces, which mechanically activate stress-sensitive ion channels/gates and alter membrane potential and capacitance.

### Cavitation (Figure 2B)

Cavitation is the production of microbubbles under the application of high frequency US in liquid or liquid-like media. Cavitation can be generally classified as non-inertial cavitation in which microbubbles remain stable and dynamic and inertial cavitation in which bubble collapse and motion are initiated by the inertia of the liquid. The occurrence of such phenomenon was multifactorial and determined by the intensity, frequency, temperature, DC, and existence of gas (Rezayat et al., 2011). Experimental results showed that non-inertial cavitation could affect cell membrane potential. As in the NICE model, cavitation may also be involved in the process. Under mechanical forces, intramembrane microbubbles are formed by the deformation of the bilayer lipid membrane, which is another medium that influences capacitive current (Krasovitski et al., 2011). In contrast, inertial cavitation can cause the marked impairment of local tissue. Typically, during HIFU, *de novo* microbubbles are associated with drastic thermal effects. The rupture of bubbles aggravated the irreversible destruction of local tissue (Izadifar et al., 2017). This effect could be beneficial to eradicate certain targets; nevertheless, it has negative consequences in the HIFU modulation process. However, inertial cavitation was rarely reported during LIFU modulation.

### Acoustic Radiative Forces (ARFs) (Figure 2C)

Acoustic radiative forces (ARFs) are a physical phenomenon that occur when an acoustic wave encounters an obstacle along its path. The acoustic energy can be converted into mechanical momentum. ARF is proposed to be one of the major non-thermal bioeffects of US. As widely acknowledged, ARFs primarily affect neural activity in two ways: the mechanical activation of stress-sensitive ion gates and channels and the alteration of membranous potential and capacitance (Rezayat and Toostani, 2016). Study showed that LIFU could activate voltage-gated sodium and calcium channels, as well as potassium channels pores without temperature elevation (Kubanek et al., 2016), indicating other mechanisms like mechanical sensitivity account for neural activation. More recently, Kubanek et al. (2016) found that when thermosensitive ion channels are knocked out in the nematode *C. elegans*, these animals still respond to LIFU. However, *C. elegans* with MS channels knocked out cannot respond to LIFU. This supports the theory that mechanical activation might be a major effect of low-intensity UNM. In addition, the results of the NICE model proposed by Plaksin et al. (2016) showed that LIFU could alter membrane capacitance, which was generated from the deformation of lipid bilayers. This mechanoelectric effect can be districted by the piezoelectric influence of acoustic waves, which is the occurrence of electric potential in piezoelectric material when mechanical stress is applied (Sassaroli and Vykhodtseva, 2016). Nonetheless, no direct evidence has been obtained, and the detailed mechanisms remain vague, further research is warranted.

Undoubtedly, ARFs are non-cavitational, non-thermal mechanisms of ultrasonic bioeffects. As ARFs are considered a promising impetus, potential fields of US are being vigorously explored. In recent studies on UNM, Ibsen et al. (2015) suggested that ARFs explained the US-treatment induced locomotive changes observed in *C. elegans* with the misexpression of transient receptor potential type 4 (TRP-4) channels in neurons. These findings provide an innovative method of cell type-specific UNM, indicating the intriguing potential of ARFs in modulating neural activity.

## Sonogenetics

To date, LIFUS has been gradually used from cell culture to clinical treatment, especially for the ultrasound-mediated neuromodulation of the central and peripheral nervous systems (Tyler et al., 2008). Even though great progress has been made in UNM and high spatial resolution can be obtained by some advanced devices, the lack of cell-specific selectivity still remains a major problem. Moreover, UNM can be influenced by acoustic intensity levels and produce unwanted heating, so secondary approaches may be applied to improve the influence of UNM. Sonogenetics, which makes neurons sensitive to acoustic neuromodulation through the expression of ion channel receptors, might show sharper spatial focus and/or deeper penetration than optogenetics (Fregni and Pascual-Leone, 2007) and better temporal resolution than magnetogenetics (Lee and Lozano, 2018). Sonogenetics has emerging as a promising novel non-invasive approach for cellular specific neural modulation.

Although many studies have proven that sonogenetics is effective, there has been controversy about its mechanism. As mentioned earlier, these mechanisms include thermal effects, cavitation, ARFs, ion channel permeability alteration and membrane deformation, while the switch control of MS ion channels through the activation of MS proteins is regarded as the mainstream theory (Maresca et al., 2018). MS ion channels are currently classified into stretch-sensitive channels, displacement-sensitive channels, and shear stress–sensitive ion channels (Morris, 1990). MS proteins will undergo a distortion when US is applied, which will activate ion channels by catalyzing conformational change (Johns, 2002; Sukharev and Corey, 2004). Although MS proteins are widely expressed in cells, only few have been found to be useful in the practice of sonogenetics, including Piezo 1, MEC-4, TRP-4, MscL, the K2p family and some VGCs (Table 3).

**TABLE 3:** Current sonogenetics studies on MS proteins.

MS protein family	Study	MS protein(s)	Ion selectivity	Target species	Ultrasound parameters	Neuromodulation	Associated mechanism	Conclusion
TRP channel	Ibsen et al. (2015)	TRP-4, a TRPN subfamily channel	Mechano-gated non-selective cation transduction	*C. elegans*	Single 10-ms, 2.25 MHz sine wave ultrasound with peak negative pressure of 0–0.9 MPa	Excitation	US-microbubble amplification	TRP-4 might be activated in response to US with peak negative pressure levels and modifies neurons by increasing microbubbles, resulting in the modulation of *C. elegans* behavior
MS channel	Ye et al. (2018)	MscL, a 3-Ns homotetrameric or homopentameric MS channel	Mechano-gated non-selective molecules: calcein efflux	Rat hippocampal CA1/CA3 neurons	Ultrasound transducer frequency:29.92 MHz Peak negative pressure: 0.12–0.45 Pa PRF: 1, 5, 10 Hz Duration: 50, 100, 200, 300, 400 ms	Excitation	Force-from-lipid theory	I92L mutant of MscL channel can be opened to control neuronal activities by using US to change membrane mechanical sensitivity
	Babakhanian et al. (2018)			*In vitro* proteoliposome model	Ultrasound transducer frequency: 0.5 MHz LIFU PRF: 1 kHz DC: 60%, 80% and CW Duration: 0, 5, 10 and 20 min	/	MscL can inhibit pore formation through LIFU modulation and membrane perturbation independent of channel gating	Compared with another two voltage-gated channels (KvAP and NaK2K F92A), the MscL channel can inhibit pore formation through LIFU modulation and membrane perturbation independent of channel gating
	Zhang et al. (2012) and Song et al. (2017)	TtMscS, a 2-Ns homotetrameric or homopentameric MS channel in *T. tengcongensis*	Mechano-gated anion over cation influx	*T. tengcongensis*	/	/	/	/
	Martinac et al. (1987)	EcMscS, a 2-Ns homotetrameric or homopentameric MS channel in *E. coli*	Mechano-gated anion influx	*E. coli*	/	/	/	/
DEG/ENaCs/ASIC channel	Kubanek et al. (2018)	MEC-4	Non-voltage-gated Na^+^ influx	*C. elegans*	Ultrasound transducer frequency: 10 MHz Peak negative pressure: 0-1 MPa PRF: 30, 100, 300, 1k, 3k, 10 kHz DC: 5, 10, 25, 50, 75, 100% Duration: 50, 100, 200, 300, 400 ms	Excitation	Cavitation, oscillations in the incident tissue and acoustic radiation forces	Ultrasound can stimulate neurons via MEC-4-dependent MS ion channels rather than via a thermal effect
VGCs channel	Tyler et al. (2008)	Nav and Cav	Nav: Voltage-gated Na^+^ influx Cav: voltage-gated Ca2^+^ influx	*Ex vivo* brains	Ultrasound transducer frequency: 0.44–0.67 MHz LIFU IPA: 2.9 W/cm^2^ ITA: 23 mW/cm^2^ PRF: 0–100 Hz	Excitation	Channel gating	US can remotely modulate brain circuit activity by activating voltage-gated sodium and calcium channels
	Kubanek et al. (2016)	Nav	Voltage-gated Na^+^ influx	Xenopus oocytes	Ultrasound transducer frequency: 10 MHz Peak negative pressure: 425 kPa–1.75 MPa PRF: 1 kHz DC: 5% Duration: 50 μs	Excitation	Channel gating	US may lead to excitation in cells that predominantly express Na^+^ US-sensitive ion channels
K2P channel	Kubanek et al. (2016)	TREK-1, TREK-2, TRAAK	Mechano-gated K^+^ efflux at −70, −10, +50 mV of membrane voltage	Xenopus oocytes	Ultrasound transducer frequency: 10 MHz Peak negative pressure: 120 kPa–240 kPa Duration: 0, 1, 2 s	Inhibition	Channel gating	US may inhibit cells that predominantly express K^+^ US-sensitive ion channels
Piezo channel	Qiu et al. (2019)	Piezo1	Mechano-gated cation: Ca2^+^ influx	*In vitro* mouse neuronal cells	Ultrasound transducer frequency: 500 kHz LIFU Peak negative pressure: 0.1, 0.3, 0.5MPa PRF: 1 kHz DC: 40% Duration: 20 min	Excitation	Channel gating	Activation of primary neurons *in vitro* can be reduced through the inhibition of Piezo1 activity stimulated by low-frequency US without microbubbles
	Liao et al. (2019)			*In vitro* HEK293T cells	Ultrasound transducer frequency: 30 MHz vertically deployed surface acoustic wave (VD-SAW) Peak negative pressure: 1.6 MPa PRF: 2 Hz, 200 Hz DC: 20% Duration: 60 s	/	/	/

### TRP-4

Transient receptor potential (TRP) was first described in 1975 as a response to photoexcitation in a *Drosophila melanogaster* mutant, and TRP channels were later found in nearly all eukaryotes (Nilius and Voets, 2005) and were recognized as promising candidates for mechanotransduction channels. In humans, TRP channels are weakly voltage sensitive and largely non-selective; cation channels are mainly classified as classical TRPs (TRPCs), no mechanoreceptor potential C TRPs (TRPNs), vanilloid receptor TRPs (TRPVs), melastatin or long TRPs (TRPMs), mucolipins (TRPMLs), polycystins (TRPPs) and ankyrin transmembrane protein (TRPA) (Christensen and Corey, 2007). TRP-4, a TRPN subfamily channel, was previously identified as a stretch-sensitive, pore-forming cation MS channel in *C. elegans* neurons (Kang et al., 2010). In the peripheral and central nervous systems, TRPs are involved in hypoxia-induced neurite growth, receptor signaling and excitotoxic cell apoptosis regulated by temperature, pressure, inflammatory agents and receptor activation (Moran et al., 2004).

Ibsen et al. (2015) firstly found that animals lacking the TRP-4 MS ion channel had notably decreased sensitivity to US-microbubble stimulation. Furthermore, the abnormal expression of TRP-4 in specific neurons might promote the neural activity of neurons expressing the TRP-4 channel upon US-microbubble stimulation and induce animal behaviors. These findings indicate that the TRP-4 MS ion channel plays an important part in the mechanism of US stimulation and that US combined with the TRP-4 MS ion channel might be used to manipulate specific neuronal functions and precise behaviors (Ibsen et al., 2015). This is the first study which combined US modulation with genetics, and the so called was proposed.

### MscL

Mechanosensitive channel with very large conductance (MscL) is a 3-nS (nanoSiemen) homotetrameric, homopentameric or homohexamer MS channel that was proved to be essential in osmoregulation (Sukharev et al., 1994, 1999). The MscL channel is a non-selective channel localized in the inner membrane and allows the passage of molecules less than 10 KD in diameter (Sukharev et al., 1994). MscL is highly conserved and ubiquitous in microbes and archaea (Kloda and Martinac, 2002; Kung et al., 2010; Boulos, 2013). It is thought to function as an osmotic emergency release valve, and its biophysics, genetics, and structures are all well characterized. Through releasing the high swelling pressure generated by the cytoplasmic permeate, MscL opens to protect bacteria from membrane damage (Sukharev et al., 1994; Tyler et al., 2008).

After succeeding in expressing MscL in RPE cells, Heureaux et al. (2014) used localized stress caused by ATC to stimulate the transfected cells in 2014. The researchers then observed the opening of MscL on RPE cells and found that ATC-induced MscL activation depends on the functional connection of the microvesicles to the intact actin cytoskeleton. Soloperto et al. (2018) first demonstrated that the functional expression of engineered MscL induced neuronal activity upon mechanical stimulation independently. These two novel studies explored the possibility of applying US to neuromodulation through MscL.

Later, research was conducted to evaluate the reaction of MscL on neural cells to US. Ye et al., 2018) succeeded in expressing MscL neurons in a primary culture and demonstrated that the transfected cells could be activated when US with low pressure was applied. The gain-of-function mutation of MscL, I92L, which is more sensitive to acoustic stimulation, achieved action potentials at a much lower peak negative ultrasonic pressure. The most recent study further investigated the activation of the MscL channel with *in vivo* stimuli and explored the best US spatiotemporal parameter for inducing such stimuli. Babakhanian et al. (2018) exploited LIFU stimulation on MscL utilizing a simplified *in vitro* proteoliposome model, which suggests that LIFU can reconstitute the MscL MS channel by modulating the cell membrane and allowing efflux through pores without full channel gating. Overall, although there exists much doubt about mechanism, MscL-based sonogenetics may offer a practicable means for neurons to non-invasively control neural activity.

Moreover, according to the force-from-lipid principle (tether proteins excluded), MscL can be activated in any membrane independent of other proteins or ligands, including liposomes (Teng et al., 2015). In other words, MscL retains its mechanosensitivity even when it is reconstituted into lipid bilayers. This means that reconstituting purified MscL into liposomes for functional expression confers the capacity to transport small molecules to liposomes. Functional MscL recombined into lipids has been proposed for application in vesicular-based drug release (Heureaux et al., 2014).

### MscS

In contrast to MscL, MS channel with very small conductance (MscS) is a 1-nS homotetrameric or homopentameric MS channel that senses membrane tension and protects cells from lysis by releasing osmolytes. The common of MscS channel includes seven portals and a β-barrel. Several MscS proteins have been identified, mainly including six from *E. coli* (EcMscS, MscK, YbdG, YnaI, YjeP, and YbiO) and three from other bacterial species (TtMscS from *Thermoanaerobacter tengcongensis*, MscSP from *Silicibacter pomeroyi*, MscCG from *Corynebacterium glutamicum*). Although there exists much dispute about mechanism of MscS channel gating with bilayer forces, Reddy et al. (2019) suggested that the interactions of the hook and pore lipid with the revised location of the lipid–protein interface play a key role in mechanotransduction between the important regions of channel (TM2 and TM3a) and the lipid–protein interface.

Most of these MscS superfamily members have a preference for cations (Wilson et al., 2013). However, EcMscS (from *Escherichia coli*) (Driscoll and Chalfie, 1991) and TtMscS (from *Thermoanaerobacter tengcongensis*) (Zhang et al., 2012) are both more selective for anions than for cations, and other MscS-like proteins tend to be cation selective (Wilson et al., 2013). A previous study showed β-barrel participates in conferring anion selectivity of TtMscS as a “selective filter” (Zhang et al., 2012).

Although no experiment had used MscS in sonogenetics, it is worth noting that one group succeeded in identifying the determinant of its anion selectivity: the β-barrel. The results of that study demonstrated that key point mutations in the β-barrel could reverse the anion selectivity of TtMscS (Zhang et al., 2012; Song et al., 2017). This means that we can control anion selectivity by changing the β-barrel. Tyler et al. (2008) and Kubanek et al. (2016) have applied sonogenetics in Xenopus oocytes resulting in excitation of neural activity by inducing Na+ and Ca2+ influx. On the contrary, Kubanek et al. (2016) explicated US to activate K2P channels and lead to an outward, hyperpolarizing K^+^ current resulting in inhibition of neural activity. According to this, it’s reasonable to suppose that excitation or inhibition of neural activity is dependent on influx or efflux of cations over anions. If we can make full use of this discovery and utilize it in sonogenetics, we may be able to activate or inhibit neural activity by letting cations or anions into neurons through manually modified MscS.

### MEC-4

Mec-4 was described as a gene that encodes the MS ion channel in TRNs (Driscoll and Chalfie, 1991). In the 1990s, extensive research on mec-4 initiated molecular studies on MEC-4, which was finally established as a 768-amino acid protein that constitutes the core subunit of the sensory mechanotransmission complex that mediates tactile sensation in *C. elegans* (Brown et al., 2007). MEC-4 belongs to a large family of ion channel proteins, which are collectively termed DEG/ENaC/ASICs and are expressed in the epithelium and neurons to form MS Na+ channels (Brown et al., 2007). Highly expressed exclusively in TRNs, MEC-4 is an essential part of the MS ion channel activated by mechanical stimuli applied to the skin of the animal. Through research on the electrophysiologic characterization of degenerins, MEC-4 combined with other DEG/ENaC/ASC members was found to sense body touch and induce the necrotic death of neurons.

Kubanek initially showed that the wild-type *C. elegans* were able to react to US. Later, experiments found that mutants that lack the ability to sense tiny temperature changes (< 0.05°C) were still able to sense US and that other mutants unable to react to mechanical stimulation showed no significant modulation of behavior after ultrasonic stimulation. The contrast between the two groups showed that the responses of the worms to US were closely related to the ability of mechanosensation, indicating that it is mechanical stimulation (especially the expression of the MS protein MEC-4), rather than thermal stimulation (Kubanek et al., 2018).

### The K2P Family

The concept of the K2P family was proposed in 1995, and Ketchum et al. (1995) predicted a new family of potassium channel proteins with two pore domains in tandem from *Saccharomyces cerevisiae*. Over the next several decades, K2P channels were divided into two P-domains in a weakly inward rectifying K+ channel (TWIK), TWIK-related K+ (TREK) channel, TWIK-related acid-sensitive K+ (TASK) channel, TWIK-related alkaline-sensitive K+ (TALK) channel, tandem pore domain halothane-inhibited K+ channel (THIK) and TWIK-related spinal cord K+ (TRESK) channel on the basis of similar structures and functions. All mammalian K2P channel subunits have unique features, including two pore domains, four transmembrane domains and an extracellular cap (Feliciangeli et al., 2015). The three channels (TREK-1, TREK-2, TRAAK) are robustly MS K2P family members that are activated when mechanical force and the opening of MS K+ channels tends to hyperpolarize cells and reduce the excitability of neurons (Kanda et al., 2019).

TREK1, TREK2, and TRAAK are MS channels from the K2P family expressed in the mammalian nervous system and play key roles in neuroprotection, pain and depression (Cadaveira-Mosquera et al., 2011). The successful expression of TREK-1, TREK-2, and TRAAK in *Xenopus oocytes* led to robust and repeatable transmembrane currents flowing through the ion channels when US was applied, which were greatly suppressed in the presence of specific channel blockers (Kubanek et al., 2016). Additionally, the increase in the concentration of extracellular K+ made the effect more obvious. All of these studies proved that US can activate transfected *Xenopus oocytes* by inducing the opening of K2P channels (Kubanek et al., 2016).

### Piezo

Piezos, including Piezo1 and Piezo2, were first identified in mammalian cells to induce MA currents in cell types (Coste et al., 2010). Piezo proteins are pore-forming subunits of ion channels that open when mechanical stimulation is applied to the membrane, thus allowing cations, such as calcium, to flow into the cell and then increase neural activity. In contrast to other ion channels, Piezos have a unique structure of four-fold repeats of 6-transmembrane units without pore-containing or repetitive domains (Coste et al., 2010). Piezo1 channels have constitutive activity after reconstitution in asymmetrical bilayers, suggesting that residual tension in the bilayer may be sufficient to open Piezo1 (Coste et al., 2012). In contrast to Piezo1 which is a polymodal sensor of diverse mechanical forces, Piezo2 can be more narrowly adjusted to specifically detect mechanical touch. The current hypothesis of Piezo channels gating is descripted that Piezo’s membrane curved into a spherical dome is regulated depending on the applied membrane tension to enable slight tension sensitivity to open channels (Kaestner and Egee, 2018).

Piezo1 is expressed in brain-derived human neural stem/progenitor cells, and its activation is involved in neurogenesis and enhanced astrogenesis. Piezo2 is strongly expressed in DRG neurons involved in sensing light touch and proprioception (Coste et al., 2010). Meanwhile, many studies about the effect of US on Piezo1 have been conducted. In one study, Piezo1 was deliberately expressed in CHO cells and HEK cells. After stimulation with US (43 MHz, 50 or 90 W/cm^2^, continuous wave), transfected cells were activated, and the transmembrane current was recorded. However, differences were observed when cells were at various height levels, which was explained by radiation force. Another study successfully utilized 30 MHz FUS generated from a vertically deployed surface acoustic wave platform to activate Piezo1-transfected HEK293T cells (Liao et al., 2019). Two other studies demonstrated that Piezo1 could be activated by high frequency US in non-neuronal cells (Gao et al., 2017; Pan et al., 2018).

Most recently, Qiu et al. (2019) developed a customized *in vitro* US stimulation system with a calcium imaging system to explore whether US could affect the function and activation of neurons through the Piezo1 channel. LIFU (500 kHz center frequency) without microbubbles could activate mouse primary neurons and induce Ca2+ ion flux into cells by gating the Piezo1 channel. This study has offered the possibility of applying neuromodulation through the Piezo1 channel *in vivo*, which could further improve sonogenetics.

### VGCs

Voltage-gated channels (VGCs) are specialized voltage-dependent pore-forming channels expressed on membranes. More than 40 years ago, it was reported that VGCs are produced by changes in protein structure in response to changes in the potential field across the cellular membrane, which is accomplished by the movement of specialized charged portions of the channel (Schneider and Chandler, 1973). The primary subfamilies of VGCs include calcium (Ca+), potassium (K+), sodium (Na+), calcium-activated potassium and HCN gated channels, which hang together to constitute physiological and pathological mechanoelectrical feedback in the body (Beyder et al., 2010; Schild and Kunze, 2012). Compared with MscL, VGCs could exhibit lower threshold mechanosensitivity when mechanical forces are converted to electrical signals (Schmidt et al., 2012). VGCs participate in electrical signaling in various tissues and cells and modulate cell excitation and proliferation, hormonal secretion, blood pressure regulation, etc. (Pinton et al., 2010).

In the nervous system, all VGCs have the potential to make their gating kinetics sensitive to transient changes in lipid bilayer tension directly via neurotransmitters and/or indirectly via intracellular second messenger systems (Sukharev and Corey, 2004). In previous studies, the influx and efflux of potassium and calcium were shown to be influenced by US regardless of US frequency, intensity and exposure time (Mortimer and Dyson, 1988; Lin et al., 2019).

In Tyler’s experiment, low-intensity and low-frequency US (LILFU, frequency is between 0.44 and 0.67 MHz, IPA = 2.9 W/cm^2^) was used to stimulate hippocampal slice cultures and *ex vivo* mouse brains. The results illustrate that LILFU can stimulate electrical neural activity by activating voltage-gated sodium and calcium channels resulting in SNARE-mediated exocytosis and synaptic transmission in hippocampal circuits. This not only shows that US can excite neurons but also shows that VGCs are active at the same time (Tyler et al., 2008). As mentioned earlier, Kubanek et al. (2016) divided Nav1.5 channels with voltage steps of −90, −70, −50, −30, −10, +10, and +30 mV in Xenopus oocytes into four groups according to whether US and Nav1.5 channel blockers were used. The study finally demonstrated that US can mediate cellular excitation through the activation of Na^+^ channels.

## Summary

UNM, as a non-invasive approach with high spatial resolution, has attracted much attention from experts in the past decades and might be a novel approach for neural modulation in the future. Sonogenetics, which combines the UNM and genetics, might further improve the spatial specificity of UNM and achieve target specific at molecular level. UNM and sonogenetics might be the optimal approach for neural modulation in the new era. However, much more studies are warranted to further corroborate the efficiency and safety of UNM and sonogenetics and optimize the parameter settings before widely clinical use.

## Author Contributions

HJ, DZ, and SW designed the review. WM and ZR drafted and revised the manuscript under the supervision of SW and DZ. BL, TZ, HC, ZW, and BH contributed to the manuscript editing. All authors contributed to the article and approved the submitted version.

## Conflict of Interest

The authors declare that the research was conducted in the absence of any commercial or financial relationships that could be construed as a potential conflict of interest.

## Abbreviations

AIUM: The American Institute of Ultrasound in Medicine
ARF: acoustic radiative force
ATC: acoustic tweezing cytometry
DC: duty cycle
DRG: dorsal root ganglion
EEG: electroencephalography
FF: fundamental frequency
FUS: focused ultrasound
HCN: hyperpolarization-activated cyclic-nucleotide
HIFU: high-intensity FUS
I: intensity
I_*SPPA*_: spatial-peak pulse-average intensity
I_*SPTA*_: spatial-peak temporal-average intensity
K2P: two-pore-domain potassium
LIFU: low-intensity FUS
LILFU: low-intensity and low-frequency ultrasound
MA: mechanically activated
MS: mechanosensitive
MscL: mechanosensitive channel with very large conductance
MscS: mechanosensitive channel with very small conductance
NICE: neuronal intramembrane cavitation excitation
nS: nanoSiemen
PRF: pulse repetition frequency
RPE: retinal pigment epithelial
TRN: touch receptor neuron
TRP: transient receptor potential
UNM: ultrasonic neural modulation
US: ultrasound
VGC: voltage-gated channel.
